# Non-Ionic Surfactant Effects on Innate Pluronic 188 Behavior: Interactions, and Physicochemical and Biocompatibility Studies

**DOI:** 10.3390/ijms232213814

**Published:** 2022-11-10

**Authors:** Orestis Kontogiannis, Dimitrios Selianitis, Diego Romano Perinelli, Giulia Bonacucina, Natassa Pippa, Maria Gazouli, Stergios Pispas

**Affiliations:** 1 Department of Basic Medical Science, Laboratory of Biology, School of Medicine National and Kapodistrian, University of Athens, 11527 Athens, Greece; 2 Theoretical and Physical Chemistry Institute, National Hellenic Research Foundation, 48 Vassileos Constantinou Avenue, 11635 Athens, Greece; 3 School of Pharmacy, Chemistry Interdisciplinary Project (CHIP), University of Camerino, Via Madonna delle Carceri, 62032 Camerino, Italy; 4 Section of Pharmaceutical Technology, Department of Pharmacy, School of Health Sciences, National and Kapodistrian University of Athens, Panepistimioupolis Zografou, 15771 Athens, Greece; 5 2nd Department of Radiology, Medical School, National and Kapodistrian University of Athens, General University Hospital Attikon, 12462 Athens, Greece

**Keywords:** Pluronic 188, non-ionic surfactants, microDSC, light scattering, MTT assay, thin-film hydration

## Abstract

The aim of this research was to prepare novel block copolymer-surfactant hybrid nanosystems using the triblock copolymer Pluronic 188, along with surfactants of different hydrophilic to lipophilic balance (HLB ratio—which indicates the degree to which a surfactant is hydrophilic or hydrophobic) and thermotropic behavior. The surfactants used were of non-ionic nature, of which Tween 80^®^ and Brij 58^®^ were more hydrophilic, while Span 40^®^ and Span 60^®^ were more hydrophobic. Each surfactant has unique innate thermal properties and an affinity towards Pluronic 188. The nanosystems were formulated through mixing the pluronic with the surfactants at three different ratios, namely 90:10, 80:20, and 50:50, using the thin-film hydration technique and keeping the pluronic concentration constant. The physicochemical characteristics of the prepared nanosystems were evaluated using various light scattering techniques, while their thermotropic behavior was characterized via microDSC and high-resolution ultrasound spectroscopy. Microenvironmental parameters were attained through the use of fluorescence spectroscopy, while the cytotoxicity of the nanocarriers was studied in vitro. The results indicate that the combination of Pluronic 188 with the above surfactants was able to produce hybrid homogeneous nanoparticle populations of adequately small diameters. The different surfactants had a clear effect on physicochemical parameters such as the size, hydrodynamic diameter, and polydispersity index of the final formulation. The mixing of surfactants with the pluronic clearly changed its thermotropic behavior and thermal transition temperature (Tm) and highlighted the specific interactions that occurred between the different materials, as well as the effect of increasing the surfactant concentration on inherent polymer characteristics and behavior. The formulated nanosystems were found to be mostly of minimal toxicity. The obtained results demonstrate that the thin-film hydration method can be used for the formulation of pluronic-surfactant hybrid nanoparticles, which in turn exhibit favorable characteristics in terms of their possible use in drug delivery applications. This investigation can be used as a road map for the selection of an appropriate nanosystem as a novel vehicle for drug delivery.

## 1. Introduction

Block copolymers are categorized as a group of macromolecules comprising of two or more chemically distinct polymerized sequences of monomers, linked together via covalent bonds. Depending on the number of individual polymers comprising a block copolymer, the latter can be called a diblock copolymer, a triblock copolymer, or a star block copolymer (A_n_B_m_ represents the simplest diblock copolymer, consisting of n units of monomer A and m units of monomer B). These molecules can be of amphiphilic nature since each polymeric “block” may have a different affinity towards aqueous solvents, due to its individual chemical structure. Such characteristics result in microphase separation on a molecular scale (5–100 nm), resulting in the production of various complex morphological nanostructures (micelles, vesicles, niosomes, cylinders, bicontinuous gyroids, etc.) via the self-assembly process, with each system obtaining distinct internal conformation [1,2,3,4,5]. This process, initiated via the minimization of the “available” to interact with the water surface area of the hydrophobic parts of the polymeric chain, can be exploited also in a supramolecular way—without the use of covalent bonds—producing even more complex nanostructures, resulting from hybrid systems of block copolymers combined with other biomaterials, such as surfactants. For this reason, amongst others, block copolymers are being studied and utilized as drug delivery carriers and innovative excipients in the pharmaceutical/nanomedicinal field. They are able to produce highly organized nanostructures, with chemically reactive, functionalized surfaces (offering the potential for the attachment of multiple probes/ligands), and they have the ability to encapsulate both hydrophilic and lipophilic APIs in sufficient quantities, exhibiting an artificially induced biomimetic internal compartmentalization [6,7,8,9]. The non-ionic triblock copolymers of the Pluronic family (namely poloxamers, or superonics) have been greatly studied, holding a plethora of promising biomedical applications in the drug delivery sector, especially due to their ability to solubilize compounds of high lipophilicity, low water solubility and bioavailability, and high toxicity (e.g., paclitaxel, doxorubicin, methotrexate, and other chemotherapeutics of BSC class II & IV), which are considered to be compounds of high risk in the medical field. Poloxamers are synthetic in nature and of the water-dispersible triblock copolymer type of poly(ethylene oxide)-b-poly(propylene oxide)-b-poly(ethylene oxide) (PEO-PPO-PEO), with more hydrophilic members, such as Poloxamer 188 (*Pluronic 188*), consisting of higher PEO/PPO—or hydrophilic/hydrophobic—chain length ratios. They are generally classified as safe and biocompatible materials [10].

Surfactants can be either naturally occurring or synthetic and exhibit great promise both in increasing the solubility of poorly dissolved agents and as functional components of drug delivery systems. Surfactants, as molecules that are amphiphilic in nature, consist of two distinct structural parts: a hydrophilic head group and a lipophilic tail (either single or double chain). Depending on other characteristics, such as the HLB ratio (hydrophilic to lipophilic balance) and their charge (ionic, non-ionic molecules), they can be categorized into different groups [11,12]. As excipients, the minimization of the surface tension between a delivery platform and the cellular membranes enhances cellular adhesion and permeability, while creating colloidal systems/dispersions of increased stability and monomeric compatibility, since a molecule of lower molecular weight can be more easily absorbed by the outer particle hydrophilic membrane [10]. The incorporation of surfactant non-ionic molecules is advantageous, since the absence of charge helps augment the circulation time, acts more protectively towards the interaction with innate plasma proteins (due to particle corona formation), and suppresses macrophage recognition [13].

In many instances, it has been established that mixtures of block copolymers with surfactants exhibit enhanced properties in drug delivery applications, resulting in hybrid systems of nanoscale sizes that protect against renal excretion and immune system recognition. Depending on the nature of the surfactant used, systems of large hydrophobic cores exhibiting high cargo loading capacities can be formulated. Block copolymer/surfactant hybrid nanosystems can present better biodistribution, drug release, toxicity, and internalization profiles when compared with conventional macro-molecular or low molecular drug carriers. Their physicochemical characteristics and thermotropic behavior can be highly tunable and their size, shape, and inner structure are highly versatile properties, which can be tailor-made with respect to each application [13].

Thin-film hydration (a physical method as opposed to chemical ones) has been established as a practical and simple technique for nanomicelle production, which is usually preferred over dialysis, direct dissolution, and self-assembly solvent-evaporation methods due to its relative reproducibility in producing particles of small diameters and high uniformity (i.e., narrow size distribution) [14].

Multiple techniques can be employed for the physicochemical, morphological, and thermal evaluation of hybrid block copolymer/surfactant nanosystems. Dynamic light scattering and static light scattering techniques (DLS, SLS), can be utilized for the evaluation of the hydrodynamic diameter, size distribution, scattering intensity, and radius of gyration of the prepared hybrid systems. Thermal analysis techniques such as micro-differential scanning calorimetry (mDSC) can reveal the thermotropic behavior of nanosystems, providing valuable information on occurring endothermic processes such as crystallization and micellization, along with the temperature range and variance at which these processes take place. Other techniques can be employed in order to examine characteristics such as micro-compressibility and sample density along with temperature increase, acting in a complementary/reassessing manner. The implementation of such characterization techniques can be conducted concurrently with the utilization of multiple thermodynamic equations, which aim to provide further insights into the biomolecular and biochemical nature of such block copolymer/surfactant interactions, such as the surfactant packing parameter:

Surfactant packing parameter = *v*_0_/*a_e_* ∗ *l*_0_

*v*_0_: volume; *a_e_*: surface of hydrophobic core; *l*_0_: length of surfactant tail.

This helps translate and presume more complete assumptions regarding the surfactant’s impact on physicochemical characteristics such as size (as expressed by hydrodynamic and gyroscopic radius). Additionally, the Laplace equation can be used in order to access the measured thermal behavior of such nanosystems through high-resolution ultrasound spectroscopy analysis, correlating sound propagation with the hybrid nanosystem density and adiabatic compressibility:

Laplace equation: *U* = 1/*√ β*_s_ ∗ *ρ*,

*β_s_*: adiabatic compressibility; *ρ*: density.

The aim of this investigation is to study the block copolymer/surfactant hybrid nanosystems containing different non-ionic surfactant molecules of small molecular weight that hold different HLB ratios and thermal transition temperatures (Tm), under aqueous solvent conditions. Polysorbate 80 (Tween 80^®^) (Tm ≈ 60 °C), Sorbitan Monopalmitate (Span 40^®^) (Tm ≈ 40 °C), Sorbitan stearate (Span 60^®^) (Tm ≈ 63 °C), and Polyethylene glycol hexadecyl ether (Brij 58^®^) (Tm ≈ 40 °C) were used as surfactants and combined with the non-ionic, amphiphilic triblock copolymer Poloxamer 188. The hybrid nanosystems were prepared via the thin-film hydration method [15]. The surfactant molecules were combined with Poloxamer 188 at three different weight ratios (90:10, 80:20, and 50:50 Poloxamer 188 to surfactant), and the prepared systems were compared in terms of their physicochemical and thermotropic characteristics with a pure Poloxamer 188 sample (100:0). The physicochemical analysis was conducted using light scattering techniques, thermal analyses were conducted using microDSC and high-resolution ultrasound spectroscopy, while microenvironmental parameters, such as micropolarity and microfluidity, were assessed through fluorescence spectroscopy. Lastly, an in vitro MTT assay using HEK293 cells was performed in order to investigate the cytotoxicity of the nanosystems at various concentrations. To the best of the authors’ knowledge, this study marks the first investigation where block copolymer Poloxamer 188 has been combined with any of these non-ionic surfactant molecules for the formation of novel block copolymer/surfactant nanosystems through the thin-film hydration technique.

## 2. Results and Discussion

### 2.1. Physicochemical Characterization of Poloxamer 188/Surfactant Nanosystems

Pure Poloxamer 188 and Poloxamer 188/surfactant hybrid nanosystems were prepared using the thin-film hydration method. The dynamic light scattering technique was employed to investigate the alteration of the innate characteristics of Poloxamer 188 nanoassemblies, which resulted from the addition of surfactants at various weight ratios (90:10, 80:20, 50:50).

**Figure 1:**
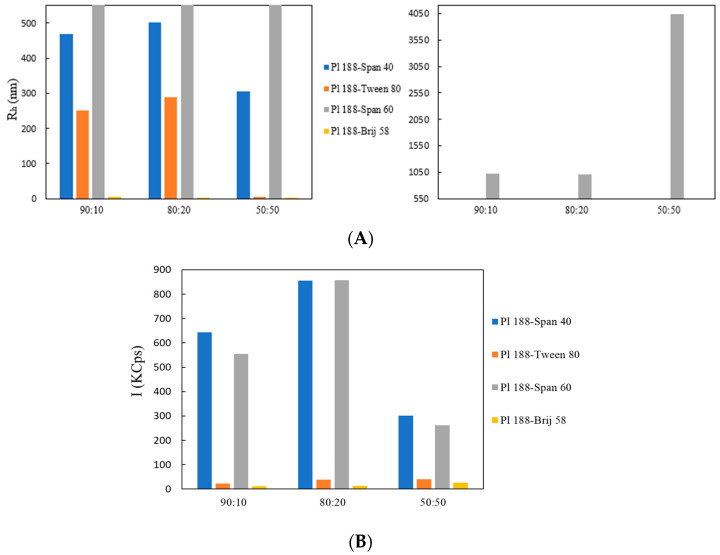
(**A**) Hydrodynamic diameter R_h_ (nm) and (**B**) Scattering Intensity, I (KCps) of Poloxamer 188-Span 40^®^ (blue bars); Poloxamer-Tween 80^®^ (orange bars); Poloxamer-Span 60^®^ (grey bars); and Poloxamer-Brij 58 (yellow bars) at different weight ratios.

Information regarding the size along with the scattering intensity (directly associated with the particle mass) are shown in Figure 1. In all experiments, the nanosystems resulting from weight ratios 50:50 Poloxamer 188 to surfactant showed the smallest sizes, with the exception of the Poloxamer 188-Span 60^®^ nanosystem. A larger particle size was observed in the case of the Poloxamer 188-Span 60^®^ mixtures (Table 1 and Figure 1) at all analyzed weight ratios, which is in accordance with existing observations and our assumptions, since Span 60^®^ contains a highly hydrophobic (C_18_) fatty acid chain, making it the most hydrophobic surfactant [16]. The stronger hydrophobicity of Span 60^®^ in comparison to Span 40^®^, which affected particle size, might be somewhat attributed to the surfactants’ chain lengths, even though they both share many similarities in their chemical structure. According to the surfactant packing parameter, the length of the hydrophobic tail influences the surfactant’s ionic character, which in turn has an effect on the packing parameter and the aggregate size (see Section 1) [17].

Poloxamer 188 represents one of the most hydrophilic members of the poloxamer group (HLB = 29), with its hydrophobic chains (PPO) forming the inner hydrophobic core of the amphiphilic micellar structure (adapted in aqueous solutions). Therefore, it is supposed that the incorporation of surfactants with lower HLB ratios (Span 60 and Span 40) results in the formation of a larger inner core, thereby affecting the total size of the micelles. The smaller sizes of certain formulations can be also attributed to the lowest polymeric ratio, since Poloxamer 188 is of the largest molar weight [18]. Surfactants with higher HLB ratios (Tween 80 and Brij 58), could co-assemble with the outer PEO corona and, especially when employed at larger ratios, could result in the formation of stronger intra-vesicular forces, leading to smaller R_g_ radii (radius of gyration) and ultimately smaller particle sizes [19].

**Figure 2:**
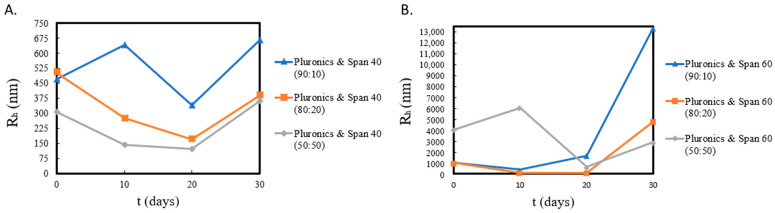
Stability assessment of (**A**) Poloxamer-Span 40^®^ and (**B**) Poloxamer-Span 60^®^ formulations through R_h_ measurements over time from day of preparation.

In certain cases, it has been reported that hybrid Poloxamer-surfactant nanosystems with large hydrophobic cores promote the formation of aggregates in aqueous media, which in turn can lead to the development of repulsive forces between the aggregates, eventually breaking down the larger particles into hybrid smaller ones (Figure 2, Figures S1 and S2) [20].

In terms of the scattering intensity, the results exhibit that the mixtures Poloxamer 188-Span 40^®^ 80:20 and Poloxamer 188-Span 60^®^ 80:20 showed increased values when compared with the pure Poloxamer 188 dispersion. Greater values are indicative of larger particle mass, but are also dependent upon particle size (proportional to D_h_^6^, where 6 is the 6th power of the particle diameter); thus, larger particles will affect the values of I (KCps), since they will result in a disproportionate scattering in comparison with smaller particles [21]. These findings are in accordance with the effect that particle size and morphology have on scattering intensity, since nanosystems of smaller diameters such as Poloxamer-Tween 80^®^ 50:50, Poloxamer 188-Brij 58^®^ 90:10, and Poloxamer 188-Brij 58^®^ 50:50, all with sizes in the 10^1^ nm range, exhibit low scattering intensity values. Morphological characteristics attained from static light scattering measurements showed a radius of gyration R_g_ value of 203 nm for the Poloxamer 188-Span 60^®^ 90:10 ratio, and an R_g_/R_h_ value equal to 0.323 (R_g_/R_h_ value is sensitive to the shape of the nanoparticles). A relatively high R_g_ value is to be expected since the size is related to the high proportion of the Poloxamer 188. In addition, the low Span 60^®^ content, which would contribute to the formation of a larger hydrophobic core (which is expected to be solvent-free), is in accordance with the R_g_ value being significantly lower than the R_h_, since in this system, the core is indeed much smaller than the hydrophilic PEO’s strongly hydrated corona. Generally, R_g_/R_h_ ratios ≤ 0.75 might indicate that the nanosystems tend to resemble hard uniform spheres, although there have been cases where nanosystems exhibiting values less than 0.75 were confirmed to be polymeric micelles via cryo-TEM imaging [22,23,24]. Generally speaking, R_g_/R_h_ values are sensitive to the radial mass density distribution of the particles.

Lastly, formulations containing Span 60^®^ and Span 40^®^ tend to be less transparent over time—due to their dilution in aqueous media—and such a result will have an impact on I values. In these formulations, the nanosystems containing the greatest surfactant ratios (50:50) exhibited the lowest I value.

Size distribution graphs, in relation to surfactant ratios and time (during a 30-day stability assessment), are included in Figures S1–S3. In terms of size distribution, it appears that the use of hydrophilic surfactants at different weight ratios achieved a slight decrease in the overall size, while the use of surfactants with lower HLB ratios resulted in particles of larger diameters. The nanosystems comprising of Poloxamer 188 and more hydrophilic surfactants tended to result in more homogeneous formulations upon increasing surfactant weight ratio, with the 50:50 ratios exhibiting the lowest PDI values (Table 1, Figure S3). The opposite results appeared with the use of more hydrophobic surfactants, with weight ratios of 90:10 Poloxamer 188 to surfactant exhibiting the lowest polydispersity.

From these results, we can conclude that the type of the incorporated surfactant, differentiated by its HLB balance and (Tm), plays a key role in characteristics such as the size, the size distribution, and most likely the morphology of the final nanosystem, since this is implied by the values of I. In all nanosystems, we should keep in mind that the solvent used was water for injection, and as such, nanosystems containing larger hydrophobic regions were more prone to molecular aggregation. In Figure S1, it is apparent how this tendency may have affected particle size over time (especially in larger nanosystems with greater surfactant ratios—Figure S1c), along with the observation that the Poloxamer 188-Span 40^®^ and Poloxamer 188-Span 60^®^ nanosystems were less transparent by the 30th day than the rest of the samples, even though the same storage conditions were applied [25,26].

### 2.2. Fluorescence Spectroscopy Results

Fluorescence spectroscopy measurements were conducted at a fixed temperature of 25 °C, a temperature lower than the transition temperature of the pure Poloxamer 188 and of the formulated nanosystems, in order to extract qualitative information regarding the internal structure and microenvironment of the nanosystems in aqueous media. The I_1_/I_3_ ratio in the fluorescence emission spectrum of pyrene and the I_excimer_/I_1_ ratio in the emission spectrum of dipyrene (hydrophobic probes) were used in order to measure the microenvironment polarity surrounding the two probes, as well as microfluidity characteristics. In aqueous media, the I_1_/I_3_ ratio was found to be between 0.85 and 1.33, while the I_excimer_/I_1_ (peak shifted to 470 nm) ratio provided values between 0.03 and 0.99, as shown in Table 2. In all nanosystems, a decrease in the values of I_1_/I_3_ was observed following an increase in the surfactant ratio, except for the Poloxamer 188-Brij 58^®^ systems. On the other hand, nanosystems containing higher Poloxamer 188 ratios tended to exhibit higher I_ex_/I_1_ values. This phenomenon might be attributed to the increase in hydrophobicity that the addition of the amphiphilic surfactants offers to a more hydrophilic Poloxamer 188 (HLB = 29) nanosystem.

In Figure S4, it is evident that the nanosystems resulting from the more hydrophilic surfactants exhibited higher excimer formation, which in turn is indicative of a higher total concentration of dipyrene, while Poloxamer 188-Span 40^®^ systems exhibited the lowest excimer intensities [27].

**Figure 3:**
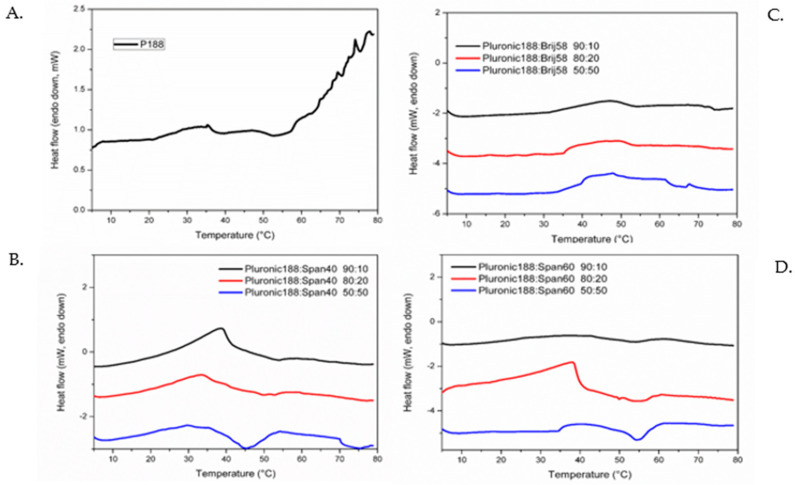
microDSC thermograms for (**A**) Poloxamer 188 (10 mg/mL), (**B**) Poloxamer 188-Span 40^®^, (**C**) Poloxamer 188-Brij 58^®^, and (**D**) Poloxamer 188-Span 60^®^ at different weight ratios.

The Poloxamer 188-Span 60^®^ formulations exhibited the lowest I_ex_/I_1_ values, which can be explained by the fact that those nanosystems have the highest transition temperatures in comparison with the other Poloxamer 188-surfactant mixtures (Figure 3). In addition, as stated earlier, Span 60^®^ represents the most hydrophobic surfactant, unable to interact with water molecules via van der Walls forces, which might result in less polar nanosystems (Table 2).

Generally, the augmentation of micropolarity (lower I_1_/I_3_ values) can be expected with the addition of surfactants with higher HLB ratios, while the formulations that exhibited increased microviscosity (decreased excimer formation) were Poloxamer 188-Span 60^®^ and Poloxamer 188-Span 40^®^. Greater excimer formation is indicative, thus, of larger microfluidity in the nanosystems’ inner domains, since it results from a greater incorporation of the hydrophobic probe into the nanosystems hydrophobic domains. Amongst all formulations, Poloxamer 188-Brij 58^®^ exhibited the greatest microfluidity (Table 2, Figure S4). Information regarding the viscosity of the nanosystems can be valuable for the estimation of the critical aggregation concentration (CAC), above which the binding between surfactant molecules and the poloxamer are cooperative, drastically changing characteristics such as the surface tension and conductivity [28].

Based on the results from Figure S4, it is interesting to witness that the excimer formation is greater for all nanosystems at the weight ratio of 50:50 Poloxamer 188 to surfactant, a property that is strongly linked with the formation of the first micelles around the critical micelle concentration (cmc). As a result of the hydrophobicity of the probes used, excimer formation below the cmc level is quite inefficient, and thus an assessment could be made through these results regarding the extraction of a cmc value. Since, at this ratio, probe molecules were found inside the nanosystems (indicative of the greater excimer formation—a larger peak in the intensity diagram), this means that the cmc is approximately at this ratio, since at surfactant concentrations well above the cmc, it would be quite unlikely to find more than one probe molecule inside each nanoparticle or hydrophobic domain. It is important to stress that in this scenario, this peak (Figure S4) does not coincide with the exact cmc, but rather can be used as a “window” around it [17]. In retrospect, the results of a cryo-TEM analysis on the morphological characteristics of the nanosystems remain to back up our hypothesis based on the preliminary spectroscopic results. In applications using ABA block copolymers of the Poloxamer family, when considering cmc, in terms of surfactant molecule’s ability to form micelles, one must keep in mind the clouding point, which represents the temperature above which phase separation begins and the hydrogen bonds at the hydrophilic head groups are broken due to increased surfactant activity. Such phenomena related to poor solubility are to be further examined via the thermal characterization of the nanosystems, where the temperature increase during microDSC characterization can be used for the cmc evaluation [29]. It is important to note that the cmc values of Poloxamer 188 reported from various publications are quite diverse, a phenomenon that might be attributed to the method used for cmc determination (surface tension vs. solubilization/dye methods), as well as to possible differences regarding the purity/grade of the triblock copolymer used [30].

### 2.3. Thermal Characterization by MicroDSC

**Figure 4:**
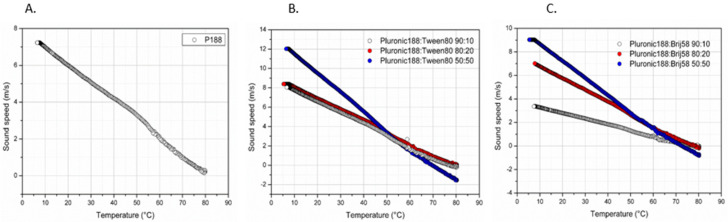
Sound speed vs. temperature for (**A**) Pl 188 (10 mg/mL), (**B**) Pl 188-Tween 80^®^, and (**C**) Pl 188-Brij 58^®^ at different weight ratios.

After obtaining essential information regarding the physicochemical characteristics of the nanosystems, the thermotropic behaviors of the Poloxamer 188-surfactant hybrid structures were determined via microDSC and high-resolution ultrasound spectroscopy analysis. The microDSC graphs are illustrated in Figure 3, and the ultrasound spectroscopic results in Figure 4. The calculated transition ™ temperatures and enthalpy values of all nanosystems are summarized in Table 3. Like all previous experiments, the thermal characteristics of the pure Poloxamer 188 sample were examined as a reference to study the thermal transition of the hybrid systems. Its T_m_ was found to be approximately 56 °C according to both techniques, while the enthalpy associated to the transition was around 0.04 J/g of solution. The observed T_m_ value for Poloxamer 188 water dispersion was in accordance with the literature: indeed, a single endothermic transition can be recognized, probably related to dehydration of the PEO-rich corona, leading to the formation of micelles or the transformation of micelles into other aggregates [30,31,32]. All hybrid samples showed an endothermic transition in the range 45–55 °C related to the Poloxamer 188 thermal transition, which is affected by the addition of the different non-ionic surfactant according to various weight ratios. Therefore, the T_m_ decreased after the introduction of the surfactant molecules to Poloxamer 188 solutions in all prepared samples.

As can be also noticed, this endothermic transition is a low energetic event for all samples with calculated enthalpy values that are comparable to that calculated for pure Poloxamer 188 dispersion [33,34]. An exception is represented by the formulations containing Span 60^®^, which at all weight ratios exhibited higher transition enthalpy values ranging from 0.135 to 0.354 J/g of solution as the ratio of Span 60^®^ increased. A pattern was observed, in which the nanosystems resulting from the combination of Poloxamer 188 with the more hydrophobic surfactants exhibited stronger thermal events (increase of the enthalpy of the transition) with an increase in the surfactant weight ratio, while the opposite phenomena took place when using the more hydrophilic surfactants, where a decrease in the proportion of Poloxamer 188 resulted in less energetic thermal interactions.

In all samples, the half-width of transition was approximately 10 °C [35]; therefore, the observed endothermic events, which began at the Tonset and ended at the Tendset, occurred roughly in the same temperature window for all systems (Figure 3 and Figure S5, Table S1), although the decrease in surfactant content did slightly increase the temperature range (Table 3) [32,36].

These results show a strong interaction between the surfactant molecules and the PPO hydrophobic chains of the triblock copolymer, which, according to previous results, are the formulations with the largest hydrodynamic radii (R_h_), scattering intensities, and possibly the greatest microviscosities.

The results obtained from mDSC were confirmed by HR-US analysis (Figure 4). The technique is based on the emission of ultrasound waves at a selective frequency in order to investigate changes in the physical properties of the Poloxamer-surfactant nanosystems related to temperature, which can be identified based on the variation of ultrasound parameters such as sound speed (m/s). From sound speed changes over temperature increase, important information regarding the thermal transitions of the formulated nanosystems can be obtained. Phase transitions due to temperature increase are accompanied by structural alterations of the nanosystems, which in turn affect the velocity of the propagation of the ultrasound waves [37]. In Table 3, the T_m_ values based on the HR-US analysis were found to be highly comparable with those from the mDSC analysis. Based on the sound speed propagation, it is evident that before and after the phase transition temperature, the values measured for each nanosystem (at all weight ratios) were different in comparison with the pure Poloxamer 188 (10 mg/mL) sample, which indicates a clear change in sample density (Figure 4). In general, the addition of surfactants in larger concentrations (50:50) exhibited the highest sound speed, which is in turn indicative that the adiabatic compressibility (β_s_) and density (ρ) of the sample have decreased (see Section 1). Adiabatic compressibility is a quantity expressing how much the pressure applied to a system can be changed without allowing for heat transfer to occur. As supported by the results shown in Figure 4, an increase in the ratio of surfactant leads to a higher sound speed change. Specifically, sound speed traces over temperature are comprised of three distinct regions in the corresponding graphs: an initial linear region, a non-linear middle part, and a final linear one. This can be explained using the previous observations, where the initial region (linear sound speed regression per temperature increase) is the effect of temperature increase in the entire sample, while the following non-linear part is the effect of polymeric desolvation (represented by a change in the curve slope), leading to the aggregation process. Finally, the second linear region indicates that the thermal process has been completed [36,38]. Lastly, it is important to underline that the results from Figure 4 show that when our samples were in the aggregate state, there was still noticeable variation in terms of sound wave propagation, which is indicative of the different structures and architectures of the aggregates formed in each case. Thus, we can logically conclude that Poloxamer 188 concentration does appear to have a noticeable effect on micelle and/or aggregate formation in terms of morphology (size, shape, and structure). Further experiments must be carried out in terms of examining the key characteristics of nanosystems, such as viscoelasticity and rheological behavior, as well as including in vitro experiments using 3D culture media in order to access how the nanosystems insert into cells.

### 2.4. In Vitro Toxicity Studies

**Figure 5:**
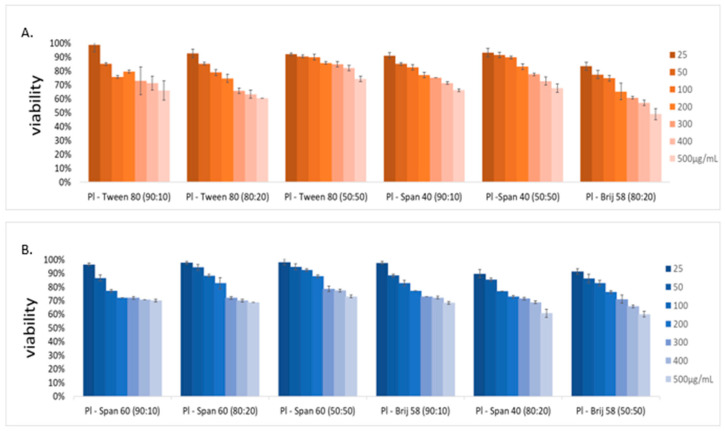
Cell viability vs. different concentrations of (**A**) Poloxamer 188-Tween 80^®^ and Poloxamer 188-Span 40^®^ and (**B**) Poloxamer 188-Span 60^®^ and Poloxamer 188-Brij 58^®^ at different weight ratios. Each graph accounts for the dispersion of the viability percentage via the standard deviation. Results after a 24-h incubation.

All formulated nanosystems were tested through in vitro experiments to investigate the cytotoxicity of the colloidal formulations. In Figure 5, cell viability (HEK293—noncancerous, initially adherent cell lines) vs. hybrid nanosystem concentration are depicted in bar diagrams. In all cases, the cells exhibited a dose-dependent toxicity. The nanosystem found to have the most toxic effects was Poloxamer 188-Brij 58^®^ 80:20, which, at the lowest concentration (25 μg/mL), achieved a cell viability rate of 83%, which was reduced down to 49% at the highest concentration (500 μg/mL). On the contrary, the other formulations of Poloxamer 188-Brij 58^®^ exhibited lower toxicity levels, since even at the highest concentration, more than two thirds of the cells remained viable.

Regarding Poloxamer 188-Tween 80^®^ nanosystems, the most toxic among them was again the same weight ratio as the Brij 58^®^ samples, exhibiting a much lower toxicity (93% cell viability at 25 μg/mL) that reduced to 60% at 500 μg/mL.

The same trend was also observed for Poloxamer 188-Span 40^®^ samples, where the highest toxicity was found in the 80:20 weight ratio, which exhibited a cell viability percentage ranging from 90% (25 μg/mL) down to 61% (500 μg/mL).

Lastly, Poloxamer 188-Span 60^®^ nanosystems appeared to have the lowest toxicity rates, regardless of the Poloxamer/surfactant weight ratio. The three formulations, namely 90:10, 80:20, and 50:50, all exhibited viability values at the lowest concentration ranging from 96% to 98% (25 μg/mL), with values dropping as the concentration increased, finally reaching values between 69% and 70% at the highest concentration (500 μg/mL).

Results using one-way ANOVA showed statistical significance between the viability percentage exhibited by the hybrid nanosystems and the control pure triblock sample, while two-way analysis (without replication), evaluating how the different surfactant ratio and concentration affected viability, again exhibited statistically significant results (*p* ≤ 0.05). The only exception to this was the Poloxamer 188-Span 60^®^ formulations, where the *p* values were slightly higher for the one-way ANOVA, exhibiting no significant difference between any of the weight ratios and the control group, but this result correlates with our previous observation that these nanosystems appear to be the least toxic (Table S2, Figure 5). Also, Poloxamer 188-Brij 58^®^ 90:10 showed no significant difference between the viability percentage of the HEK293 cells and the control group, with a *p* value equal to 0.47. It is worth mentioning that ANOVA represents a simple statistical method used to examine whether the differences, in terms of the characteristic that you want to evaluate, between two or more groups of data are statistically significant, with a lower *p*-value indicating a stronger correlation between your independent variable (which differs between the analyzed groups) and the evaluated characteristic. Based on the number of independent variables (i.e., weight ratio, surfactant type, etc.) as well as the variable type, you can proceed to choose the appropriate type of analysis (one-way ANOVA, two-way ANOVA, Factorial ANOVA, etc.) As such, a two-way analysis can be used when evaluating the effects of two independent variables on the characteristic in question (in this case, the viability percentage).

All experiments took place at 37 °C, which is a temperature much lower than the T_m_ of both the pure Poloxamer 188 and the pure surfactants (literature values) of all the formulated nanosystems [39,40,41]. As such, there was no point in examining the toxicity on an individual component level, and each time, the whole formulation was directly tested. It is important to note that a formulation cytotoxicity profile is strongly related to the cellular absorption that takes place through endocytosis, due to the small size of the nanosystems. For this reason, one must keep in mind the results of Table 1, and the fact that the larger diameter of the Poloxamer 188-Span 60^®^ nanosystems may have had an effect on cell internalization when compared with other hybrid systems. Advancing to future in vivo experimentation, special consideration must be given to mucosal adhesion in terms of size-dependent filtering of particles based on the mucosal mesh’s pores [42,43]. All nanosystems were formulated less than a week prior to the MTT analysis, and each experiment, regarding each weight ratio, was replicated twice (two distinct wells incubated at the same time point, under the same conditions). This method is based on the HEK293 cellular incubation with MTT (tetrazolium salt), a yellowish substance which under the action of the appropriate mitochondrial oxidoreductase enzymes can be reduced to formazan (purple color). If this reduction doesn’t occur (or occurs partially), it signals that the mitochondrial activity is absent (or diminished), and as such, a percentage of the cells are no longer viable. The results are shown as the mean ± standard deviation of all experiments (*n* = 2). When using the MTT assay, one must account for the fact that the intracellular metabolism of MTT has been linked with mitochondrial injury related to apoptosis events, and as such a well, used as a control (MTT, incubation substrate, HEK293 cells), was helpful in accounting for this phenomenon. [44] Lastly, the nature of the MTT assay is to detect MTT reduction as a measure of mitochondrial activity, from which secondary processes, such as cell death, is inferred. It is, however, possible that a small cellular percentage of each well is in fact quiescent rather than dead, due to metabolic stress, and thus that the results of Figure 5 may be in fact slightly lower than the actual viable cells [45].

A follow-up study could differentiate whether the effect of the nanosystems on the cells is apoptotic or necrotic, since MTT is used in order to detect cell stress through the mitochondrial metabolic activity and does not distinguish between the two.

## 3. Materials and Methods

### 3.1. Materials

**Figure 6:**
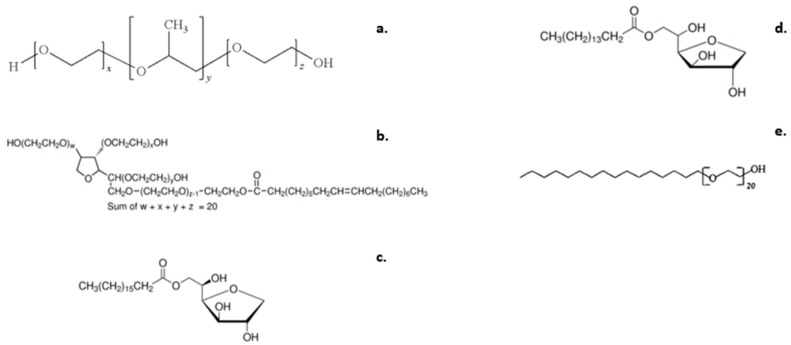
The chemical structures of (**a**) Poloxamer 188 (Tm = 56 °C), (**b**) Tween 80^®^, (**c**) Span 60^®^, (**d**) Span 40^®^, and (**e**) Brij 58^®^.

Poloxamer 188 (PLX 188, in the form of white microbeads) (Figure 6a), Tween 80^®^ (Polysorbate 80) (Figure 6b), Span 60^®^ (Sorbitan stearate) (Figure 6c), Span 40^®^ (Sorbitan Monopalmitate) (Figure 6d), and Brij 58^®^ (Polyethylene glycol hexadecyl ether) (Figure 6e) were purchased from Sigma-Aldrich (Merck Group). Analytical grade chloroform (CHCl_3_) as the organic solvent was purchased from Fisher Chemical ^TM^. HPLC-grade water (bottled, water for injection) was purchased from DEMO AΒΕΕ, Athens.

### 3.2. Methods

#### 3.2.1. Preparation of Poloxamer 188 and Poloxamer 188/Surfactant Colloidal Dispersions

The hybrid nanosystems, along with the pure Poloxamer 188 sample, were prepared using the thin-film hydration method. Namely, stock solutions of Poloxamer 188 and the surfactant molecules in organic solvent (CHCl_3_) were prepared at a concentration of 10 mg/mL. Then, mixing the appropriate volumes of the stock solutions created three distinct—in terms of weight ratio—Poloxamer 188-to-surfactant formulations (i.e., 90:10, 80:20, and 50:50 wt ratios) for each surfactant, resulting in 12 different mixed samples in total. Afterwards, each sample was transferred into a spherical flask which was inserted into the rotary evaporator (Hei-VAP series CORE-heidolph^®^) for 20 min, at a temperature between 40 °C to 50 °C, until the organic solvent had completely evaporated. Each formulation was then left to rest for a period of 24 h (at 4 °C). After this period, hydration of each prepared film took place using HPLC-grade water (DEMO^®^). Hydrophilic Millipore^®^ syringe filters were used for filtering the water before hydration. Lastly, each sample was transferred into the sonication bath following a specific protocol (3-min sonication, followed by a 2-min rest period, followed by another 2-min sonication) in order to avoid the formation of aggregates, especially in the mixtures containing more hydrophobic surfactants. The final dispersions were placed in glass vials and stored at 4 °C under refrigerated conditions.

#### 3.2.2. Light Scattering Methods

The size, size distribution, and scattering intensity of the prepared nanosystems in aqueous media were measured using dynamic light scattering (DLS) and static light scattering (SLS). The dilution protocol followed for the insertion of each formulation, along with the pure Poloxamer 188, in the sample cell was 50μL of sample in 2 mL of HPLC–grade water. The hydrodynamic radius (R_h_), size distribution (polydispersity index, PDI), and scattering intensity (I), along with the radius of gyration (R_g_)—when needed—were evaluated. All measurements were implemented at a fixed temperature (25 °C) and at a fixed scattering angle of 90° degrees. Measurements were conducted using a wide-angle light scattering photometer by ALV GmbH, CGS-3, able to perform dynamic and static light scattering experiments simultaneously. This set up comprised of a He-Ne 22 mW laser source, a compact goniometer system with an Avalanche photodiode detector interfaced with an ALV/LSE-5003 electronics unit, and an ALV-5000/EPP multi-tau digital photon correlator.

#### 3.2.3. MicroDSC Analysis and High-Resolution Acoustic Spectroscopy

MicroDSC analyses were carried out using a microcalorimeter (mDSC III, Setaram, France). All samples were loaded inside the Hastelloy cells (700 μL) and subjected to a thermal protocol including: isotherm at 5 °C for 20 min; heating ramp from 5 °C to 80 °C at 1 °C/min; cooling ramp from 80 °C to 5 °C at 1 °C/min. The peak temperature (Tm, °C), onset temperature (Tonset, °C), and enthalpy (ΔH, J/g of solution) were calculated from the peak and the area of the transition by the tangent method through the instrument software (Set soft2000, Setaram, Lyon, France). The same samples were also analyzed through high-resolution ultrasound spectroscopy (HR-US 102 high-resolution spectrometer, Ultrasonic Scientific, Dublin, Ireland) at a frequency of 5.4 MHz by applying the same thermal program used for mDSC analyses. The results are expressed as differential relative sound speed over temperature, and Tm values were calculated from the fist-derivate of the signal. All measurements were performed thrice.

#### 3.2.4. Fluorescence Spectroscopy

The fluorescence spectra were collected in order to gather information on the internal microenvironment of the prepared hybrid nanostructures, along with a pure Poloxamer 188 sample, which was used as a “baseline” for surfactant influence on microfluidity and micropolarity characteristics (NanoLog Fluorometer spectrometer by Horiba Jobin Yvon). All experiments were carried out at a fixed temperature of 25 °C, which is lower than the transition temperatures of both Poloxamer 188 and the surfactants (see Section 2.4). Each sample was analyzed twice using two hydrophobic emission probes, namely pyrene and dipyrene. The insertion protocol in the sample cell followed was 1 mL of sample mixed with 1 μL of probe. Analysis took place after a 24-h rest period (4 °C). The exact protocol is described in our previous study [19].

#### 3.2.5. In Vitro Toxicity

HEK293 cells (rounded cells which grow in suspension-liquid culture) were cultivated at a fixed temperature of 37 °C using a DMEM High Glucose culture medium (provided by BioSera) containing 10% FBS, 2 mmol/L glutamine, 100IU/mL penicillin, and 100 μg/mL streptomycin. At the (approximately) 48-h mark, the medium was replaced, and the cells were passaged on a weekly basis using the trypsin/EDTA method. After reaching a sufficient confluency, cells were transferred to a 96-well plate and 5000 cells/well were seeded. Incubation took place using a steri-cycle CO_2_ incubator (HEPA Class 100, Thermo Electron Corporation^®^). The growth medium was renewed before the start of the experiment. HEK293 cell viability was evaluated through the use of a microscope, and a healthy cell percentage was achieved (>85%). Optical assessment was also performed via possible discoloration of the 96-well plate (transition towards a more yellowish color indicated cell stress, or possible culture contamination). The different nanoformulation concentrations to which cells were exposed ranged between 25–500 μg/mL. Incubation time was set at 24-h. All samples were incubated concurrently under exact conditions. All the procedures took place in a sterile environment. All the incubations took place using sterile microplates under a sterile hood. All experiments were replicated twice (*n* = 2), while the analysis took place less than a week from the initial formulation of the nanosystems.

## 4. Conclusions

The goal of this work was to prepare hybrid nanosystems composed of Poloxamer 188 and non-ionic surfactants, namely Tween 80^®^, Span 60^®^, Span 40, and Brij 58^®^, via the thin-film hydration method. For each surfactant, different nanosystems were formulated at different Poloxamer 188/surfactant weight ratios (90:10; 80:20; 50:50). The nanosystems were evaluated through various light scattering and thermal analysis techniques, while in vitro cytotoxicity experiments were conducted. The nature of the incorporated surfactant affected the size, size distribution, and most likely the morphology of the prepared nanosystems, in comparison to pure Poloxamer 188, as implied by the scattering intensity values. The nanosystems comprising of more hydrophilic surfactants formed more homogeneous nanosystems as surfactant ratio increased, while Poloxamer 188-Span 60^®^ nanosystems presented the largest sizes at all weight ratios utilized. All systems exhibited colloidal stability at least 10 days post formulation. Systems containing surfactants with higher HLB ratios exhibited the greatest micropolarity in fluorescence spectroscopy analysis, while the Poloxamer 188-Span 60^®^ and Poloxamer 188-Span 40^®^ nanosystems showed greater microviscosity. Excimer formation was greater at weight ratios 50:50 Poloxamer 188 to surfactant, which is indicative of initial micelle formation near the cmc. All samples had an endothermic transition around 55 °C related to Poloxamer 188, while the addition of the surfactants to various weight ratios seemed to have affected this behavior. All samples exhibited a rather low transition enthalpy, indicating weak thermal events, except Poloxamer 188-Span 60^®^ formulations, which exhibited an even higher transition enthalpy as the surfactant ratio increased. Results obtained from mDSC were confirmed by further HR-US analysis, where noticeable variation in terms of sound wave propagation indicated that the different structures and architecture of the mixed micelles formed logical results from alterations to Poloxamer 188 concentration. In all cases, the cells exhibited a dose-dependent toxicity toward the prepared nanosystems. Most nanosystems exhibited higher cytotoxicity values at the 80:20 Poloxamer 188 to surfactant weight ratio. The Poloxamer 188-Span 60^®^ hybrid systems exhibited the lowest toxicity values at all weight ratios. All nanosystems displayed low toxicity, with the exception of the Poloxamer 188-Brij 58^®^ 80:20 system.

In conclusion, we used thin-film hydration for the formulation of Poloxamer 188/surfactant hybrid nanosystems, which exhibited small sizes, high homogeneity, and favorable characteristics regarding their possible utilization as novel drug delivery carriers. This investigation can be used as a road map for deeper understanding of pluronic/surfactant interactions and for the selection of appropriate combinations of such, towards the formulation of innovative, amphiphilic nanosystems of tunable physicochemical and thermal characteristics.

## Figures and Tables

**Table 1:** The physicochemical characteristics of the prepared nanosystems.

System	Weight Ratio	R_h_ (nm) ^a^	I (KCps) ^b^	PDI ^c^
Poloxamer 188	100:0	425.0	763	0.480
Poloxamer 188-Tween 80	90:10	252.0	23	0.530
Poloxamer 188-Tween 80	80:20	290.0	39	0.603
Poloxamer 188-Tween 80	50:50	6.0	23	0.435
Poloxamer 188-Span 40	90:10	470.0	644	0.520
Poloxamer 188-Span 40	80:20	503.0	855	0.560
Poloxamer 188-Span 40	50:50	305.0	302	0.530
Poloxamer 188-Span 60	90:10	1018.0	555	0.509
Poloxamer 188-Span 60	80:20	1002.0	857	0.489
Poloxamer 188-Span 60	50:50	4047.0	261	0.542
Poloxamer 188-Brij 58	90:10	5.0	11	0.582
Poloxamer 188-Brij 58	80:20	346.0	13	0.520
Poloxamer 188-Brij 58	50:50	4.0	26	0.431

^a^ R_h_(nm): Hydrodynamic diameter; ^b^ I(KCps): Scattering intensity; ^c^ PDI: polydispersity index.

**Table 2:** I_1_/I_3_ and I_ex_/I_1_ values of the prepared hybrid block copolymer-surfactant systems at a fixed temperature of 25 °C.

System	Weight Ratio	Pyrene	Dipyrene
		I_1_/I_3_	I_ex_/I_1_
Poloxamer 188	100:0	1.17	0.42
Poloxamer 188-Tween 80	90:10	1.18	0.37
Poloxamer 188-Tween 80	80:20	1.16	0.57
Poloxamer 188-Tween 80	50:50	1.08	0.27
Poloxamer 188-Span 40	90:10	1.01	0.15
Poloxamer 188-Span 40	80:20	0.95	0.12
Poloxamer 188-Span 40	50:50	0.96	0.09
Poloxamer 188-Span 60	90:10	1.21	0.03
Poloxamer 188-Span 60	80:20	0.96	0.14
Poloxamer 188-Span 60	50:50	0.85	0.12
Poloxamer 188-Brij 58	90:10	0.96	0.99
Poloxamer 188-Brij 58	80:20	1.33	0.70
Poloxamer 188-Brij 58	50:50	1.13	0.65

**Table 3:** The thermotropic characteristics of the studied systems.

System	Weight Ratio	mDSC		HR-US (Sound Speed)
		Transition Temperature (°C)	Enthalpy (J/g of Solution)	Transition Temperature (°C)
Poloxamer 188	100:0	55.66 ± 0.75	0.041 ± 0.011	56.82 ± 0.96
Poloxamer 188–Tween 80	90:10	53.69 ± 0.06	0.030 ± 0.002	54.86 ± 0.74
Poloxamer 188–Tween 80	80:20	53.80 ± 0.12	0.029 ± 0.011	53.36 ± 0.66
Poloxamer 188–Tween 80	50:50	53.18 ± 0.22	0.016 ± 0.013	52.47 ± 0.33
Poloxamer 188–Span 40	90:10	53.00 ± 0.64	0.029 ± 0.012	52.27 ± 0.97
Poloxamer 188–Span 40	80:20	52.30 ± 0.36	0.037 ± 0.009	50.35 ± 0.95
Poloxamer 188–Span 40	50:50	45.67 ± 0.46	0.124 ± 0.013	46.61 ± 0.37
Poloxamer 188–Span 60	90:10	53.20 ± 0.19	0.135 ± 0.012	54.13 ± 0.46
Poloxamer 188–Span 60	80:20	55.56 ± 0.30	0.182 ± 0.012	56.12 ± 0.53
Poloxamer 188–Span 60	50:50	54.22 ± 0.14	0.354 ± 0.010	55.17 ± 0.55
Poloxamer 188–Brij 58	90:10	53.89 ± 0.23	0.070 ± 0.009	56.86 ± 0.45
Poloxamer 188–Brij 58	80:20	53.14 ± 0.13	0.022 ± 0.007	55.66 ± 0.66
Poloxamer 188–Brij 58	50:50	52.57 ± 0.16	0.011 ± 0.005	56.77 ± 0.56

## Data Availability

Not applicable.

## Supplementary Materials

The supporting information can be downloaded at: https://www.mdpi.com/article/10.3390/ijms232213814/s1.
