# Supplementary material for: Non-Ionic Surfactant Effects on Innate Pluronic 188 Behavior: Interactions, and Physicochemical and Biocompatibility Studies
Source: Int J Mol Sci. 2022 Nov 10;23(22):13814. doi: 10.3390/ijms232213814 (PMC9697813; doi:10.3390/ijms232213814)
Supplement: Supplementary file 1 [file ijms-23-13814-s001.zip › ijms-2021770-supplementary.pdf]

Supplementary Material

Article

Non-ionic surfactant effects on innate Pluronic 188 behavior: interactions, and physicochemical and biocompatibility studies

Orestis Kontogiannis[1,2], Dimitris Selianitis[2] Diego Romano Perinelli[3], Giulia Bonacucina [3], Natassa Pippa[4], Maria Gazouli[1,5], Stergios Pispas[2,*]

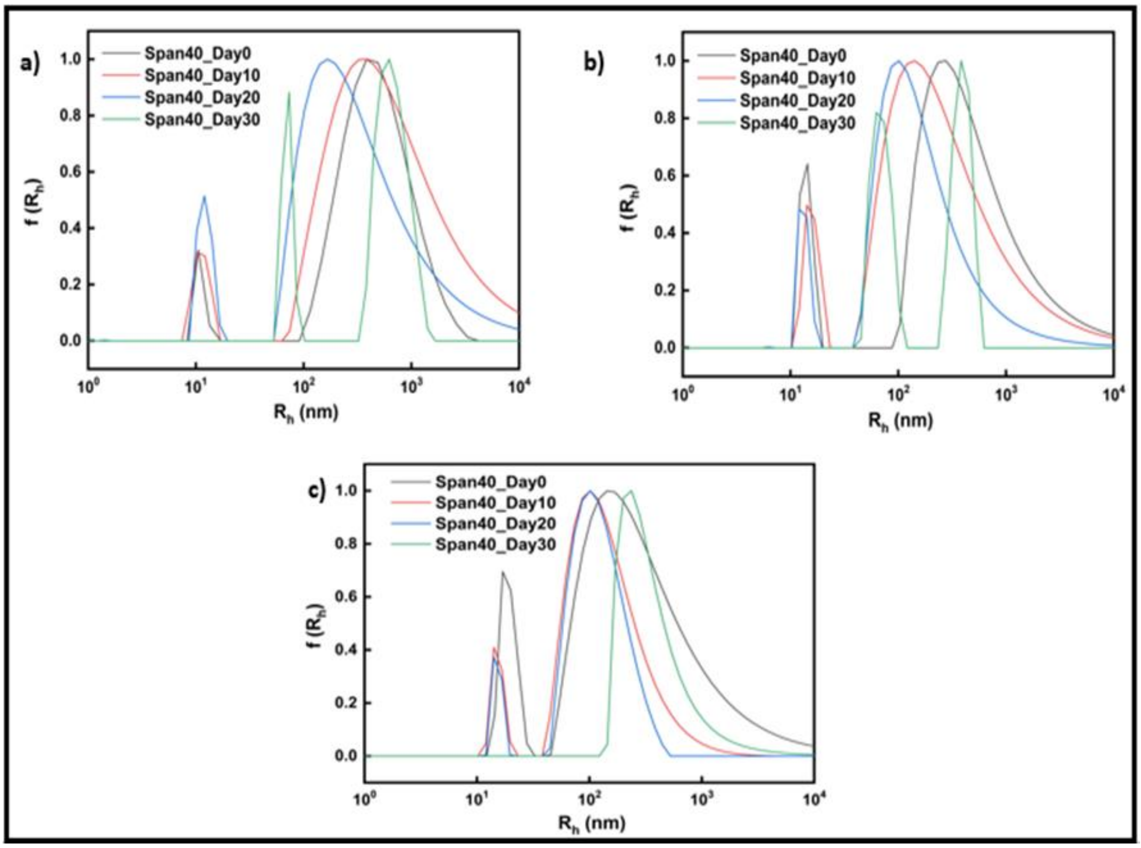

Figure S1. Comparative plots of size distributions from DLS measurements for Poloxamer 188 mixtures with Span 40® surfactant throughout a 30-day stability assessment for a) mixing ratio 90:10, b) mixing ratio 80:20 and c) mixing ratio 50:50.

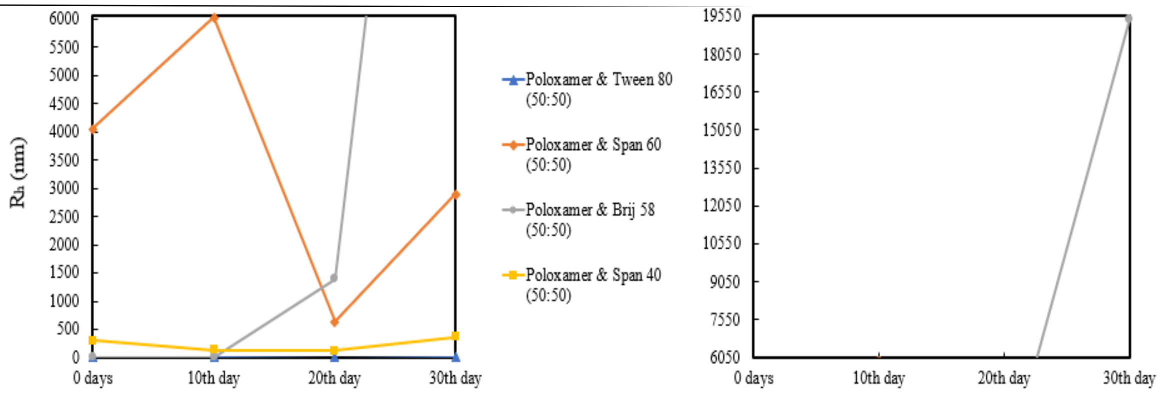

Figure S2. Stability assessment of prepared nanosystems.

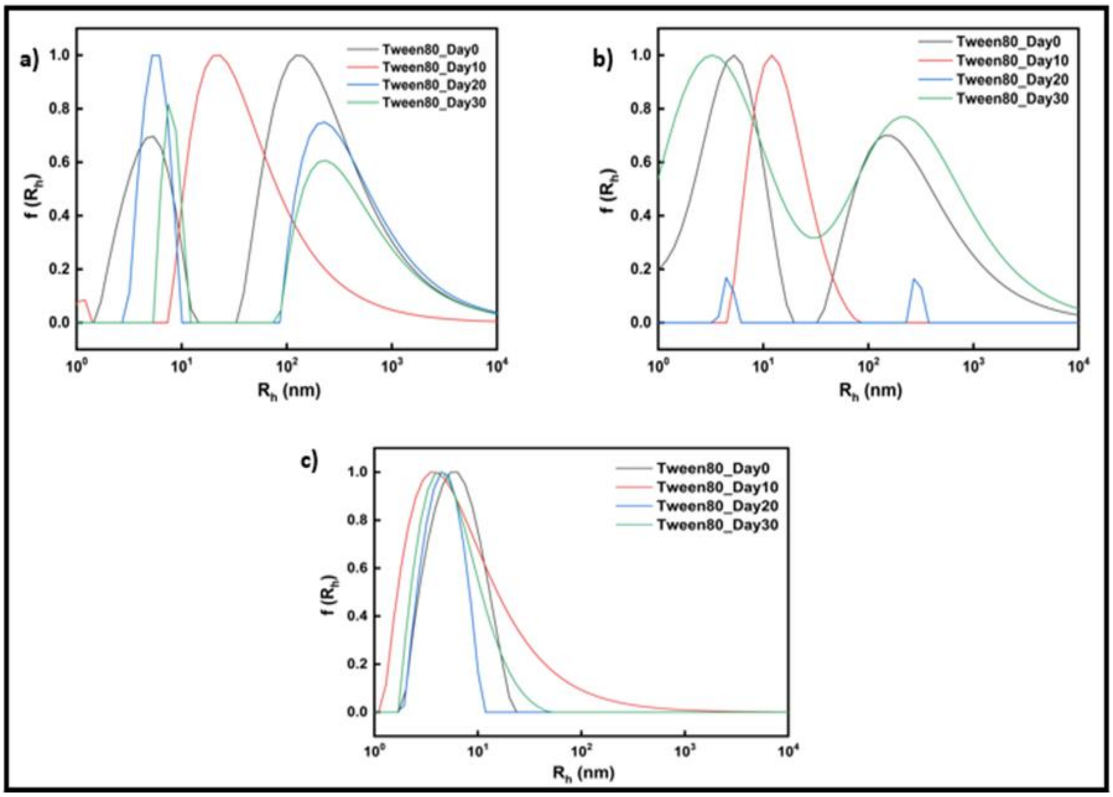

Figure S3. Comparative plots of size distributions from DLS measurements for Poloxamer 188 mixtures with Tween 80® surfactant throughout a 30-day stability assessment for a) mixing ratio 90:10, b) mixing ratio 80:20 and c) mixing ratio 50:50.

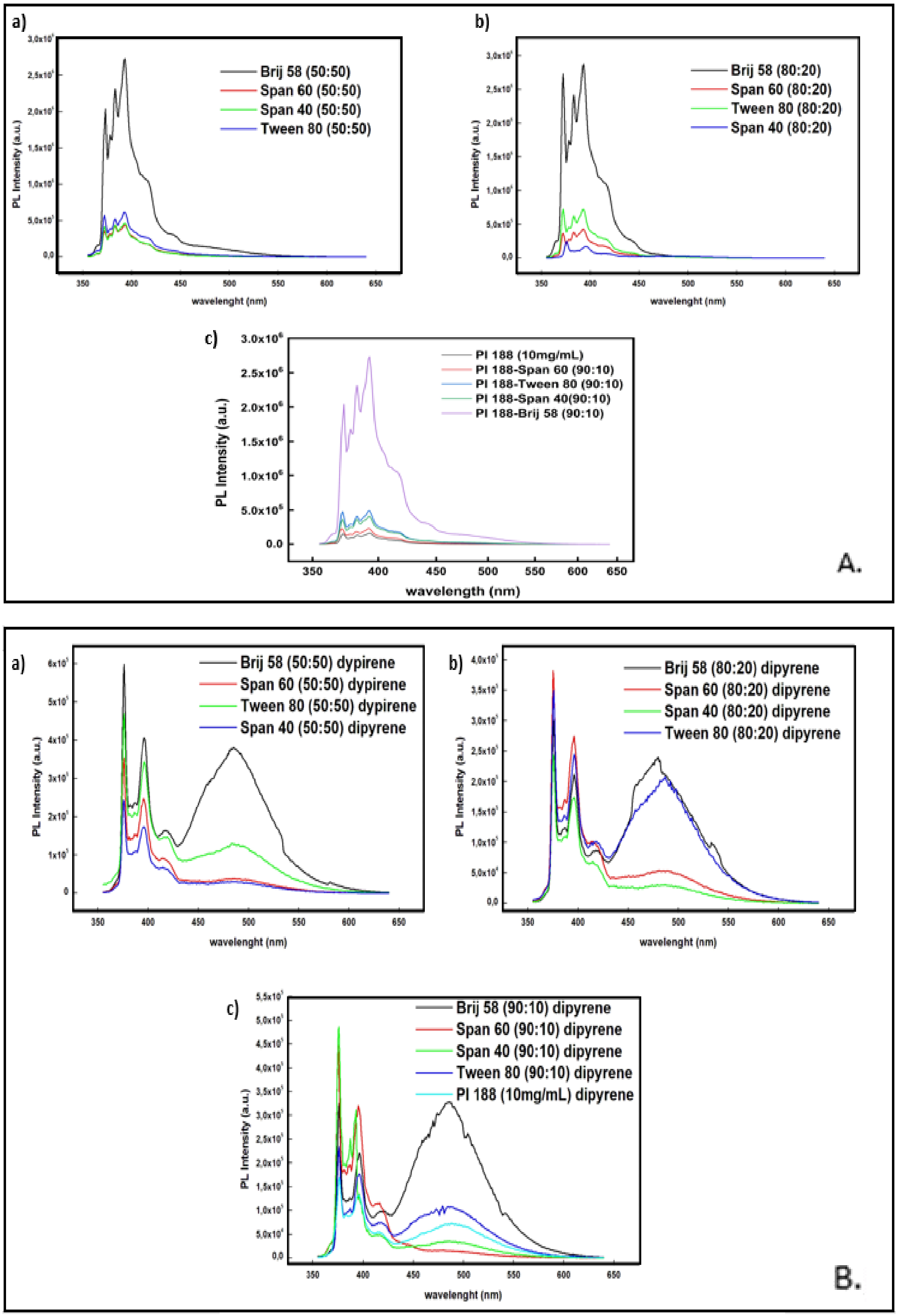

Figure S4. Comparative fluorescence spectra from different mixtures by the use of **A.** pyrene and **B.** dipyrene as fluorescence probes. Poloxamer 188-surfactant mixtures at different ratios:

a) mixing ratio of 50:50, b) mixing ratio of 80:20, and c) mixing ratio of 90:10, along with Pl 188 (10mg/mL) as control.

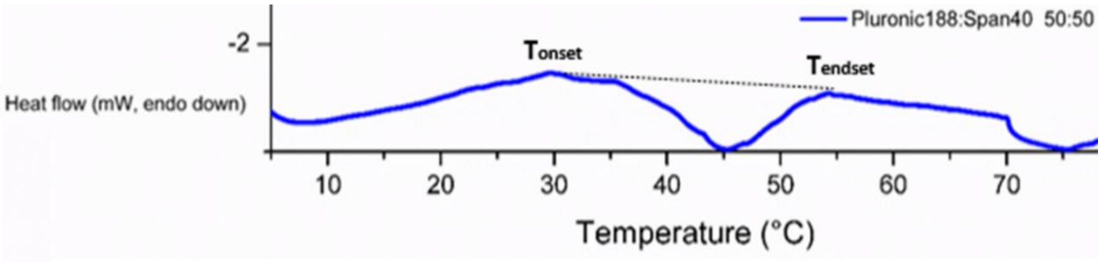

Figure S5. Temperature window where micellization occurs for Poloxamer 188-Span 40® 50:50 ratio from mDSC.

Table S1. Calculation of the Tonset (°C) and Tendset (°C) for pure Poloxamer 188 solution (C = 10mg/mL) and Poloxamer 188/surfactant mixed nanosystems.

	mDSC	
	Tonset (°C)	Tendset (°C)
Poloxamer 188	49.75±1.10	55.43±1.41
Poloxamer 188 : Tween 90:10	50.47±0.37	55.86±0.41
Poloxamer 188 : Tween 80:20	49.17±1.13	56.80±1.12
Poloxamer 188 : Tween 50:50	50.35±1.59	55.74±0.78
Poloxamer 188 : Span 40 90:10	51.18±0.12	56.11±0.99
Poloxamer 188 : Span 40 80:20	47.13±0.59	55.38±1.20
Poloxamer 188 : Span 40 50:50	39.77±1.70	53.06±1.05
Poloxamer 188 : Span 60 90:10	42.91±0.41	60.12±1.68
Poloxamer 188 : Span 60 80:20	41.10±1.83	59.44±1.17
Poloxamer 188 : Span 60 50:50	46.53±1.80	60.12±1.62
Poloxamer 188 : Brij 58 90:10	49.89±0.61	58.45±0.75
Poloxamer 188 : Brij 58 80:20	50.58±0.25	57.18±0.57
Poloxamer 188 : Brij 58 50:50	50.10±0.79	54.86±1.38

Table S2. One-way ANOVA analysis of Poloxamer 188-Span 60® 90:10 analysis in comparison with control group in terms of concentration related HEK293 cell viability ($p \geq 0.05$).

Anova: Single Factor

SUMMARY

Groups	*Count*	*Sum*	*Average*	*Variance*
Pl - Span 60 (90:10)	7	13189	1884.142857	58742.80952
control	7	14252	2036	0

ANOVA

Source of Variation	*SS*	*df*	*MS*	*F*	*P-value*	*F crit*
Between Groups	80712.07143	1	80712.07143	2,747981313	0.123269	4.747225
Within Groups	352456.8571	12	29371.40476			
Total	433168.9286	13				
